# A new species of *Hyphessobrycon* Durbin from northeastern Brazil: evidence from morphological data and DNA barcoding (Characiformes, Characidae)

**DOI:** 10.3897/zookeys.765.23157

**Published:** 2018-06-06

**Authors:** Erick Cristofore Guimarães, Pâmella Silva De Brito, Leonardo Manir Feitosa, Luís Fernando Carvalho-Costa, Felipe Polivanov Ottoni

**Affiliations:** 1 Universidade Federal do Maranhão, Programa de Pós-Graduação em Biodiversidade e Conservação. Av. dos Portugueses 1966, Cidade Universitária do Bacanga, CEP 65080-805, São Luís, MA, Brazil; 2 Universidade Federal do Maranhão, Programa de Pós-Graduação em Biodiversidade e Biotecnologia da Amazônia Legal. Av. dos Portugueses 1966, Cidade Universitária do Bacanga, CEP 65080-805, São Luís, MA, Brazil; 3 Universidade Federal de Pernambuco, Programa de Pós-Graduação em Biologia Animal. Av. Professor Moraes Rego 1235, Cidade Universitária, CEP: 50670-901, Recife, PE, Brazil; 4 Universidade Federal do Maranhão, Departamento de Biologia, Laboratório de Genética e Biologia Molecular, Av. dos Portugueses 1966, Cidade Universitária do Bacanga, CEP 65080-805, São Luís, MA, Brazil; 5 Universidade Federal do Maranhão, Laboratório de Sistemática e Ecologia de Organismos Aquáticos, Centro de Ciências Agrárias e Ambientais, Campus Universitário, CCAA, BR-222, KM 04, S/N, Boa Vista, CEP 65500-000, Chapadinha, MA, Brazil; 6 Universidade Federal do Maranhão, Programa de Pós-Graduação em Oceanografia. Av. dos Portugueses 1966, Cidade Universitária do Bacanga, CEP 65080-805, São Luís, MA, Brazil

**Keywords:** *Hyphessobryconsensu stricto*, integrative taxonomy, Pristellinae, rosy tetra clade, clado rosy tetra, *Hyphessobryconsensu stricto*, taxonomia integrativa, Pristellinae

## Abstract

A new species of *Hyphessobrycon* is described for the upper Munim and Preguiças river basins, northeastern Brazil, supported by morphological and molecular species delimitation methods. This new species belongs to the *Hyphessobrycon* *sensu stricto* group, as it has the three main diagnostic character states of this assemblage: presence of a dark brown or black blotch on the dorsal fin, absence of a black midlateral stripe on its flank and the position of Weberian apparatus upward horizontal through dorsal margin of operculum. Our phylogenetic analysis also supported the allocation of the new species in this group; however, it was not possible to recover the species sister-group. *Pristella maxillaris* and *Moenkhausia hemigrammoides* were recovered as the sister-clade of the *Hyphessobrycon* *sensu stricto* group.

## Abstract (pt)

Uma nova espécie de *Hyphessobrycon* é aqui descrita para as bacias do alto Rio Munim e Preguiças, nordeste do Brasil, sustentada por métodos morfológicos e moleculares de delimitação de espécies. Essa nova espécie é membro do grupo *Hyphessobrycon* *sensu stricto*, já que possui os três principais estados de caracteres diagnósticos desse agrupamento: presença de uma mancha marrom escura ou negra na nadadeira dorsal, ausência de uma faixa lateral no meio do flanco e a posição do aparelho de Weber localizado acima da horizontal da margem dorsal do opérculo. Nossa análise filogenética também apoia o posicionamento da nova espécie dentro desse grupo, entretanto não foi possível recuperar o grupo-irmão da espécie. *Pristella maxillaris* e *Moenkhausia hemigrammoides* foram recuperados com sendo o clado irmão do grupo *Hyphessobrycon* *sensu stricto.*

## Introduction

*Hyphessobrycon* Durbin, 1908 is one of the most species-rich genera of Characidae, currently comprising approximately 150 valid species (Ohara et al. 2017). It is widely distributed along the river basins of the Neotropical region, from southern Mexico to the La Plata River basin in northeastern Argentina (Carvalho and Malabarba 2015, Teixeira et al. 2015, Garcia-Alzate et al. 2017), with highest diversity in the Amazon basin (Miquelarena and López 2006, Lima et al. 2013, Bragança et al. 2015, Carvalho and Malabarba 2015, Marinho et al. 2016, Carvalho et al. 2017, Moreira and Lima 2017).

Extensive data show that *Hyphessobrycon* is not a monophyletic group (Carvalho et al. 2017, Moreira and Lima 2017). It was diagnosed by an artificial combination of character states proposed by Eigenmann (1917), such as: the presence of an adipose fin; maxillary with few teeth or none; lateral line incomplete; third infraorbital bone not in contact with the sensory canal of the preopercle; premaxillary with two series of teeth; and caudal-fin lobes without scales at the base. Nevertheless, new species descriptions continue to follow this artificial combination (e.g., Ohara et al. 2017, Garcia-Alzate et al. 2017, Carvalho et al. 2017, Moreira and Lima 2017).

In addition, some artificial species groups of *Hyphessobrycon* were proposed based on the combination of character states (e.g., Weitzman and Palmer 1997, Zarske 2014, García-Alzate et al. 2008a, b, 2013, Carvalho and Malabarba 2015) relying mainly on coloration patterns. However, in many cases, it is not possible to assign without reasonable doubt to which group a particular species belongs (Bragança et al. 2015).

One of this species group was termed as “group F” by Géry (1977), being defined by the presence of a dark brown or black blotch on dorsal fin and no midlateral stripe on body. This group was previously termed as “*callistus* group”, with a similar composition and definition by Géry (1961). Weitzman and Palmer (1997) proposed the name “rosy tetra clade” for this assemblage, including approximately 30 species of *Hyphessobrycon* and a few other probably closely related species belonging to other genera. These authors also confirmed the presence of the black blotch on the dorsal fin as one of the main diagnostic features of this assemblage. However, they also stated that this blotch was absent in some of its species (e.g., *H. ecuadoriensis* Eigenmann & Henn, 1914, *H. loweae* Costa & Géry, 1994 and *H. panamensis* Durbin, 1908).

After that, Carvalho (2011) and Carvalho and Malabarba (2015) proposed the group named *Hyphessobrycon* *sensu stricto*, diagnosed by the position of Weberian apparatus upward horizontal through dorsal margin of operculum, presence of a black blotch on dorsal fin and the absence of a midlateral black stripe on body, with a more restricted composition than the “rosy tetra clade” *sensu* Weitzman and Palmer (1997), comprising: *H. compressus* (Meek, 1904), *H. bentosi* Durbin, 1908, *H. copelandi* Durbin, 1908, *H. epicharis* Weitzman & Palmer, 1997, *H. eques* (Steindachner, 1882), *H. erythrostigma* (Fowler, 1943), *H. georgettae* Géry, 1961, *H. haraldschultzi* Travassos, 1960, *H. hasemani* Fowler, 1913, *H. khardinae* Zarske, 2008, *H. megalopterus* (Eigenmann, 1915), *H. micropterus* (Eigenmann, 1915), *H. minor* Durbin, 1909, *H. pulchripinnis* Ahl, 1937, *H. pyrrhonotus* Burgess, 1993, *H. rosaceus* Durbin, 1909, *H. roseus* (Géry, 1960), *H. simulatus* (Géry, 1960), *H. socolofi* Weitzman, 1977, *H. sweglesi* (Géry, 1961), *H. takasei* Géry, 1964 and *H. werneri* Géry & Uj, 1987. Other species recently referred to the “rosy tetra clade” such as *Hyphessobrycon dorsalis* Zarske, 2014, *H. jackrobertsi* Zarske, 2014, *H. paepkei* Zarske, 2014 and *H. pando* Hein, 2009 share these traits, but their taxonomic status is uncertain (Carvalho and Malabarba 2015). The key point is that the remaining species of *Hyphessobrycon* included in the other groups will probably need to be assigned to other genera or new genera (*Hyphessobrycon* *sensu lato*) (Carvalho and Malabarba 2015).

One way to overcome the confusing taxonomy of problematic groups, to have accurate species identifications and species diversity estimates of groups is to use different operational criteria for species delimitation (Goldstein and Desalle 2010, Padial et al. 2010). Any operational criteria (species delimitation methods) may separately provide evidence about the species limits and identity independently from other criteria (de Queiroz 2005, 2007), but evidence corroborated from multiple operational criteria is considered to produce stronger hypotheses of lineage divergence (de Queiroz 2007, Goldstein and Desalle 2010), converging to the proposal for an integrative taxonomy (Goldstein and Desalle 2010, Padial et al. 2010). Gathering morphological and molecular data has become a common practice to identify and delimit species of fish (Teletchea 2009), mainly in groups including cryptic or morphologically similar species. The most widespread molecular method used in taxonomy has been the DNA barcoding, which consists on the use of a single gene from mitochondrial DNA (cytochrome oxidase subunit I – COI) as a proxy for species differentiation (Hebert et al. 2003). In fact, several studies have been carried out using molecular markers and new species have been delimited and/or described, in most cases, based both on molecular and morphological evidence (e.g., Costa and Amorim 2011, Costa et al. 2012, Roxo et al 2012, Villa-Verde et al. 2012, Castro-Paz et al. 2014, Costa et al. 2014, Benzaquem et al 2015, Mattos et al. 2015, Costa et al. 2017).

A new species of *Hyphessobrycon*, member of the *Hyphessobrycon* *sensu stricto* Carvalho and Malabarba, 2015 is herein described from the Munim and Preguiças river basins, two coastal river basins of the Maranhão State, northeastern Brazil, based on both morphology and molecular data.

## Materials and methods

### Morphological analysis

Measurements and counts were made according to Fink and Weitzman (1974), with exception for the scale rows below lateral line, which were counted to the insertion of pelvic fin. Horizontal scale rows between the dorsal-fin origin and lateral line do not include the scale of the median predorsal series situated just anterior to the first dorsal-fin ray. Counts of supraneurals, vertebrae, procurrent caudal-fin rays, unbranched dorsal and anal fin rays, branchiostegal rays, gill-rakers, premaxillary, maxillary, and dentary teeth were taken only from cleared and stained paratypes (C&S), prepared according to Taylor and Van Dyke (1985). The four modified vertebrae that constitute the Weberian apparatus were not included in the vertebra counts and the fused PU1 + U1 was considered as a single element. Osteological nomenclature follows Weitzman (1962). Institutional abbreviations follow Sabaj-Pérez (2016), with addition of **CICCAA** Coleção Ictiológica do Centro de Ciências Agrárias e Ambientais and **CPUFMA** Coleção de Peixes da Universidade Federal do Maranhão.

### Comparative material examined

All specimens are from Brazil.

***Hyphessobrycon amandae*** Géry & Uj, 1987: UFRJ 1557, 5 spcms, Goiás State, Jussara municipality. ***H. bentosi***: CICCAA 00849, 2 spcms, aquarium trade. **H. cf. bentosi**: CICCAA 00701, 1 spcm, Pará State, Paragominas municipality. CICCAA 00702, 2 spcms, Pará State, Paragominas municipality. CICCAA 00703, 1 spcm (C&S), Pará State, Paragominas municipality. ***H. bifasciatus*** Ellis, 1911: UFRJ 0068, 6 spcms, Espírito Santo State, Marataízes and Guarapari municipality. ***H. copelandi***: CICCAA 00722, 2 spcms, Pará State, Marabá municipality. ***H. diancistrus*** Weitzman, 1977: UFRJ 2166, 55 spcms, Tocantins State, Ilha do Bananal municipality. ***H. eques***: CICCAA 00715, 4 spcms (C&S), Minas Gerais State, Tombos municipality. CICCAA 00710, 51 spcms, Minas Gerais State, Tombos municipality. ***H. griemi*** Hoedeman, 1957: UFRJ 4496, 7 spcms, Santa Catarina State, Esplanada municipality. ***H. haraldschultzi***: CICCAA 00873, 20 spcms, Tocantins State, Ilha do Bananal municipality. ***H. itaparicensis*** Lima & Costa, 2001: CICCAA 00314, 6 spcms, Sergipe State, Areia Branca municipality. ***Pristella maxillaris*** (Ulrey, 1894): CICCAA 00850, 2 spcms, aquarium trade. ***H. micropterus***: FMNH 57916, 1 spcm, Rio São Francisco at Lagoa do Porto (Photograph of a Holotype). CICCAA 00300, 24 spcms, Bahia; Barras municipality. CICCAA 00699, 8 spcms (C&S), Barras, Bahia municipality. ***H. reticulatus*** Ellis, 1911: UFRJ 0107, 4 spcms, Rio de Janeiro State, Desengano municipality. ***H. sergipanus*** Bragança, Ottoni & Rangel-Pereira, 2016: CICCAA 00296, 11 spcms, Sergipe State, Estância municipality. UFRJ 5582, 8 spcms, Mato Grosso State, Poconé municipality. UFRJ 3937, 4 spcms, Mato Grosso State, Cárceres municipality. ***H. stegemanni*** Géry, 1961: UFRJ 1988, 17 spcms, Tocantins State, Porto Nacional municipality. ***H. sweglesi***: CICCAA 00852, 2 spcms, trade aquarium. ***H. wernerei***: MUZUSP 42365, 1 spcm, Pará State, Santa Maria do Pará municipality. CICCAA 00751, 1 spcm, Pará State, Paragominas municipality.

### DNA extraction, amplification, and sequencing

DNA extraction was carried out with the Wizard Genomic DNA Purification kit (Promega) following manufacturer’s protocol. DNA quality was evaluated by agarose gel electrophoresis stained with GelRed (Biotium) and was quantified using Nanodrop 2000 (Thermo Fisher Scientific). DNA was stored at -20 °C until further procedures. Samples (N= 4; Table 1) were amplified using standard PCR (Polymerase Chain Reaction) for partial cytochrome oxidase subunit 1 (COI) gene, with primers designed by Ward et al. (2005) (FISHF1 5´-TCAACCAACCACAAAGACATTGGCAC-3´and FISHR1 5´-TAGACTTCTGGGTGGCCAAAGAATCA-3´). Amplification reactions were performed in a total volume of 15 µl comprising 1× buffer, 1.5 mM MgCl_2_, 200 µM dNTP, 0.2 uM of each primer, 1 U of Taq Polymerase (Invitrogen), 100 ηg of DNA template, and ultrapure water. The amplification program consisted of a denaturation of 2 min at 94 °C, followed by 35 cycles of 30s at 94 °C, 30s at 54 °C, and 1 min at 72 °C, ending in an extension phase of 10 min at 72 °C. Amplicons were visualized in 1% agarose gel electrophoresis stained with GelRed (Biotium) and purified with Illustra GFX PCR DNA and Gel Purification Kit (GE Healthcare). Samples were sequenced using both forward and reverse primers and BigDye Terminator kit 3.1 Cycle Sequencing kit (Thermo Fisher Scientific) in ABI 3730 DNA Analyser (Thermo Fisher Scientific) Consensus sequences were edited in Geneious 9.0.5 (Kearse et al. 2012) and aligned using ClustalW (Thompson 1994) with those from *Hyphessobrycon* species available in Barcode of Life Database (BOLD) and Genbank (NCBI-National Center for Biotechonology Information) (accession numbers are in Table 1).

**Table 1.** Sampling sites, specimens and DNA sequence information included in the study.

Species	Locality	Basin/drainage	Country	GenBank/BoldSystems	Catalog number
*Hyphessobrycon piorskii* sp. n.	Anapurus, Maranhão	Munin	Brazil	MF765796	CICCAA00725
*Hyphessobrycon piorskii* sp. n.	Anapurus, Maranhão	Munim	Brazil	MF765797	CICCAA00726
*Hyphessobrycon piorskii* sp. n.	Barreirinhas, Maranhão	Preguiças	Brazil	MG791915	CICCAA01650
*Hyphessobrycon piorskii* sp. n.	Anapurus, Maranhão	Munim	Brazil	MG791914	CICCAA01651
*Hyphessobrycon bentosi*	Barcelos, Amazonas	Negro	Brazil	HYP097-13	INPA37684-5939
*Hyphessobrycon bentosi*	Barcelos, Amazonas	Negro	Brazil	HYP098-13	INPA37684-5940
*Hyphessobrycon bentosi*	Barcelos, Amazonas	Negro	Brazil	HYP099-13	NPA37684-5942
*Hyphessobrycon bentosi*	Barcelos, Amazonas	Negro	Brazil	HYP100-13	INPA37684-5943
*Hyphessobrycon bentosi*	Manaus, Amazonas	–	Brazil	HYP116-13	INPA39527-BA1
*Hyphessobrycon bentosi*	Manaus, Amazonas	–	Brazil	HYP117-13	INPA39527-BA2
*Hyphessobrycon bentosi*	Manaus, Amazonas	–	Brazil	HYP118-13	INPA39527-BA3
*Hyphessobrycon bentosi*	Manaus, Amazonas	–	Brazil	HYP119-13	INPA39527-BA4
*Hyphessobrycon copelandi*	Tabatinga, Amazonas	Solimões	Brazil	HYP094-13	INPA37683-TU1
*Hyphessobrycon copelandi*	Tabatinga, Amazonas	Solimões	Brazil	HYP095-13	*INPA37683-TU1*
*Hyphessobrycon copelandi*	Tabatinga, Amazonas	Solimões	Brazil	HYP096-13	INPA37683-TU1
*Hyphessobrycon eques*	Santarém, Pará	Amazonas	Brazil	HYP070-13	INPA37678-IC2
*Hyphessobrycon eques*	Parintins, Amazonas	Amazonas	Brazil	HYP072-13	INPA37680-AL1
*Hyphessobrycon erythrostigma*	Tabatinga, Amazonas	Solimões	Brazil	HYP073-13	INPA37681-AP1
*Hyphessobrycon erythrostigma*	Tabatinga, Amazonas	Solimões	Brazil	HYP074-13	INPA37681-AP2
*Hyphessobrycon epicharis*	São Gabriel da Cachoeira, Amazonas	Negro	Brazil	HYP002-13	INPA37665-JUF1
*Hyphessobrycon epicharis*	São Gabriel da Cachoeira, Amazonas	Negro	Brazil	HYP003-13	INPA37665-JUF2
*Hyphessobrycon megalopterus*	–	–	–	FJ749058	–
*Hyphessobrycon megalopterus*	–	–	–	KU568879.1	–
*Hyphessobrycon pyrrhonotus*	Santa Isabel do rio Negro, Amazonas	Negro	Brazil	HYP039-13	INPA37672-TRO1
*Hyphessobrycon pyrrhonotus*	Santa Isabel do rio Negro, Amazonas	Negro	Brazil	HYP040-13	INPA37672-TRO10
*Hyphessobrycon pyrrhonotus*	Santa Isabel do rio Negro, Amazonas	Negro	Brazil	HYP042-13	INPA37672-TRO2
*Hyphessobrycon socolofi*	Santa Isabel do rio Negro, Amazonas	Negro	Brazil	HYP020-13	INPA37667-UR1
*Hyphessobrycon socolofi*	Santa Isabel do rio Negro, Amazonas	Negro	Brazil	HYP022-13	INPA37667-UR7
*Hyphessobrycon rosaceus*	São Gabriel da Cachoeira, Amazonas	Negro	Brazil	HYP032-13	INPA37669-MAC4
*Hyphessobrycon rosaceus*	Nova Airão, Amazonas	Negro	Brazil	HYP069-13	INPA37677-FU1
*Hyphessobrycon rosaceus*	São Gabriel da Cachoeira, Amazonas	Negro	Brazil	HYP082-13	INPA37682-ACA1
*Hyphessobrycon sweglesi*	São Gabriel da Cachoeira, Amazonas	Negro	Brazil	HYP024-13	INPA37668-JAR1
*Hyphessobrycon sweglesi*	São Gabriel da Cachoeira, Amazonas	Negro	Brazil	HYP025-13	INPA37668-JAR2
*Hyphessobrycon sweglesi*	São Gabriel da Cachoeira, Amazonas	Negro	Brazil	HYP028-13	INPA37668-JAR5
*Moenkhausia hemigrammoides*	Rupununi Road-Guyana	–	Guyana	HYP101-13	INPA38532-PR1
*Pristella maxillaris*	–	–	–	KU568982.1	–
*Pristella maxillaris*	–	–	–	KU568981.1	–
*Hyphessobrycon flammeus*	–	–	Brazil	FUPR988-09	LBPV-40464
*Hyphessobrycon anisitsi*	–	–	Brazil	GBGCA516-10	FJ749040

### Species concept, species delimitation, and diagnoses

The unified species concept (de Queiroz 2005, 2007) is herein adopted by expressing the conceptual definition shared by all traditional species concepts – “species are (segments of) separately evolving metapopulation lineages” – when operational criterion elements to delimit taxa are excluded from the concepts. According to this concept, species are treated as hypothetical and could be tested by the application of distinct criteria (species delimitation methods) (de Queiroz 2005, 2007). It allows for any criteria to separately provide evidence about species limits and identities, independently from other criteria (de Queiroz 2005, 2007). Evidence corroborated from multiple operational criteria is considered to produce stronger hypotheses of lineage separation (de Queiroz 2007, Goldstein and Desalle 2010), a practice called “integrative taxonomy” (Dayrat 2005, Goldstein and Desalle 2010, Padial et al. 2010).

Two distinct operational criteria to delimit species, based on morphological and molecular data, were implemented here. The Population Aggregation Analysis (Davis and Nixon 1992) is a character-based method (hereafter PPA), which consists of an exclusive shared combination of character states assigned to a given population or group of populations. The second method, DNA barcoding, as proposed by Hebert et al. (2003 a, b, 2004 a, b) (hereafter DBC), is a genetic distance-based cut-off method.

### Population Aggregation Analysis (PAA)

Only morphological character states were used for this method. The morphological data was based on both examined material (see Comparative material examined) and the literature (e.g., Géry 1977, Géry and Uj 1987, Costa and Géry 1994, Plaquete et al. 1996, Weitzman and Palmer 1997, Zarske 2008, Hein 2009, Carvalho 2011, Lima et al. 2013, Zarske 2014, Carvalho and Malabarba 2015, Carvalho et al. 2017). The data obtained by this method are presented in the diagnosis section of results.

### DNA barcoding (DBC)

Pairwise genetic distances between species were calculated using Kimura-2-parameters model (K2P) (Kimura 1980) on the MEGA 7 software (Tamura et al. 2011). Evolutionary relationships among sequences were reconstructed by Bayesian inference using the MrBayes (Huelsenbeck and Ronquist 2001) plugin in Geneious 9.0.5. An independent run with a chain length of 10 million, a burn-in length of 500,000 generations, and subsampling trees every 10,000 generations was carried out under the GTR (generalized time reversible) evolutionary model, which was estimated in jmodeltest (Darriba et al. 2012). *Hyphessobrycon flammeus* Myers, 1924 and *H. anisitsi* (Eigenmann, 1907) were used as outgroup. The ingroup was composed by the remaining terminals.

## Results

### Hyphessobrycon piorskii sp. n.

Taxon classificationAnimaliaCharaciformesCharacidae

http://zoobank.org/379CFBEA-C3FE-4729-9469-029732DC62BC

Figures 1 , 2

#### Holotype.

CICCAA 00695, 25.9 mm SL, Brazil, Maranhão State: stream at the Anapurus municipality, 03°40'14"S, 43°07'10"W, 05 Feb 2017, Guimarães E. C. and Brito P. S.

#### Paratypes.

All from Brazil, Maranhão State: CICCAA 00430, 15,18.4–25.2 mm SL; CICCAA 00696, 15, 19.9–24.4mm SL, CICCAA 00697, 16 (C&S) 19.3–24.5 mm SL; CICCAA 00698, 6, 1 (C&S) 22.0–20.4 mm SL; CICCAA 00750, 9, 20.0–25.3mm SL; CPUFMA 171664, 15, 19.5–23.1 mm SL; UFRJ 11553, 6, 19.1–22.1 mm SL collected with holotype. CICCAA 00089, 1 (C&S) 25.2 mm SL, stream at Mata de Itamacaoca, Chapadinha municipality; 03°44'50"S, 43°19'21"W, 02 Apr. 2016, Ottoni F. P., Oliveira E., Nascimento I., Fernandes R., Carneiro V. leg. CICCAA 00431, 21, 15.3–19.8mm SL, stream at the Anapurus municipality, 03°40'53"S, 43°07'23”W, 15 Jan. 2017, W; Aguiar R. leg. CICCAA 00881, 1, 29.4 mm SL, stream at Mata de Itamacaoca, Chapadinha municipality; 03°44'45"S, 45°19'15”W, 15 Jul. 2017, Campos D., Oliveira E., Viana S., Lopes M., Sousa R. leg. CICCAA 01563, 1, 21.6 mm SL, stream at Mata de Itamacaoca, Chapadinha municipality; 03°44'55"S 43°19'55"W, 19 Nov. 2017, Guimarães E. C., Brito P. S., Ottoni F. P., Lucas O., Sousa R. leg. CICCAA01654, 1, 26.9 mm SL, stream at the Anapurus municipality, 03°40'14"S, 43°07'10"W, 17 Jan. 2018, Guimarães E. C. and Brito P. S. leg. CICCAA 01382, 5, 22.7–27.2 mm SL, stream at Mata Fome, Barreirinhas municipality, 02°39'47"S, 42°48'16"W, 15 Jun., 2017, Guimarães E. C., Brito P. S., Ottoni F. P., Ferreira B. R. CICCAA 02008, 12 (C&S), 15.4–18.3 mm SL, stream at Mata Fome, Barreirinhas municipality; 02°39'47"S, 42°48'16"W, 15 Jun., 2017, Guimarães E. C., Brito P. S., Ottoni F. P., Ferreira B. R. leg.

#### Diagnosis

**(PAA).** The new species *Hyphessobrycon piorskii* sp. n., promptly differs from most congeners except by species of *Hyphessobrycon* *sensu stricto* by the presence of a dark brown or black blotch on dorsal fin (vs. absence), no midlateral stripe on the body (vs. presence) and Weberian apparatus upward horizontal through dorsal margin of operculum (vs. downward).

The new species herein described differs from all of its congeners from *Hyphessobrycon* *sensu stricto*, with exception to *H. bentosi* and *H. hasemani*, by possessing an inconspicuous vertically elongated humeral spot [vs. approximately rounded humeral spot in *H. copelandi*, *H. erythrostigma*, *H. jackrobertsi*, *H. minor*, *H. pando*, *H. paepkei*, *H. pyrrhonotus*, *H. roseus*, *H. socolofi*, and *H. sweglesi*; humeral spot horizontally or posteriorly elongated in *H. epicharis*, *H. khardinae*, and *H. werneri*; conspicuous humeral spot in *H. eques*, *H. haraldschultzi* Travassos, 1960, *H. micropterus*, *H. megalopterus*, *H. simulatus* and *H. takasei*; and absence of humeral spot in *H. compresus*, *H. dorsalis* Zarske, 2014, *H. georgettae*, *H. pulchripinnis*, and *H. rosaceus*].

**Figure 1.**
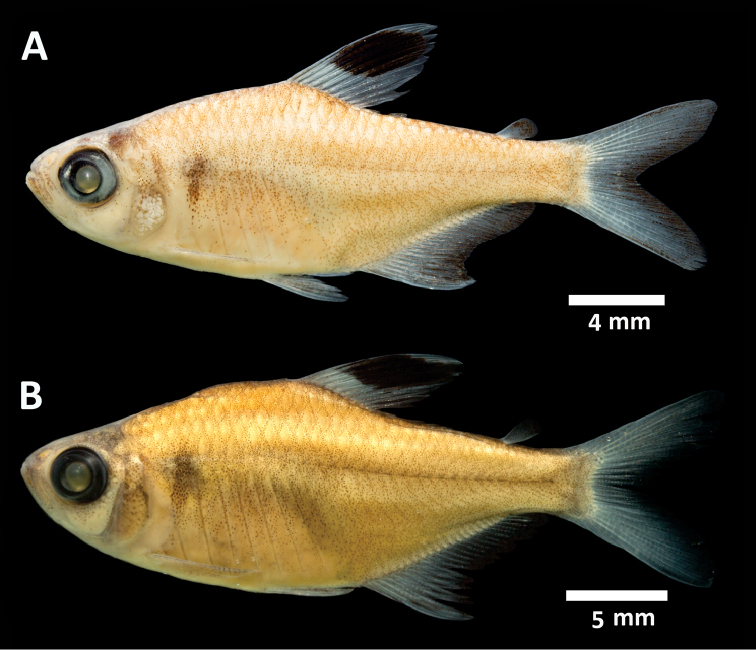
*Hyphessobrycon piorskii* sp. n. **A** CICCAA 00695, holotype, 25.9 mm SL, Brazil: Maranhão State: Munim River basin **B** CICCAA 00881, paratype, 29.4 mm SL, Brazil: Maranhão State: Munim River basin (photographed by Felipe Ottoni).

**Figure 2.**
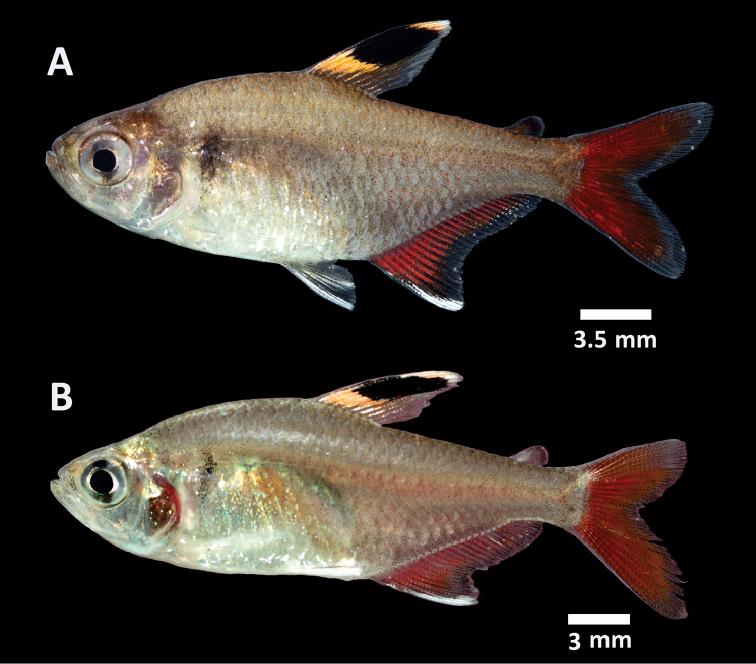
*Hyphessobrycon piorskii* sp. n. **A** CICCAA 00698, paratype, 26.9 mm SL, Brazil: Maranhão State: Munim River basin; living specimen photographed immediately after collection **B** CICCAA 00089, paratype, 25.2 mm SL, Brazil: Maranhão State: Munim River basin; living specimen photographed immediately after collection (photographed by Felipe Ottoni).

The new species differs from *H. bentosi* by the absence of an extended and pointed dorsal and anal-fin tips (Figures 1, 2) [vs. extended and pointed dorsal and anal-fin tips]; and from *H. hasemani* by the dorsal-fin black spot shape, which is located approximately at the middle of the fin’s depth, not reaching its tip [vs. extended along all the fin, reaching its tip in adults] and by presenting tri to unicuspid teeth in the inner row of premaxillary and dentary [vs. pentacuspid teeth].

#### Description.

**Figure 3.**
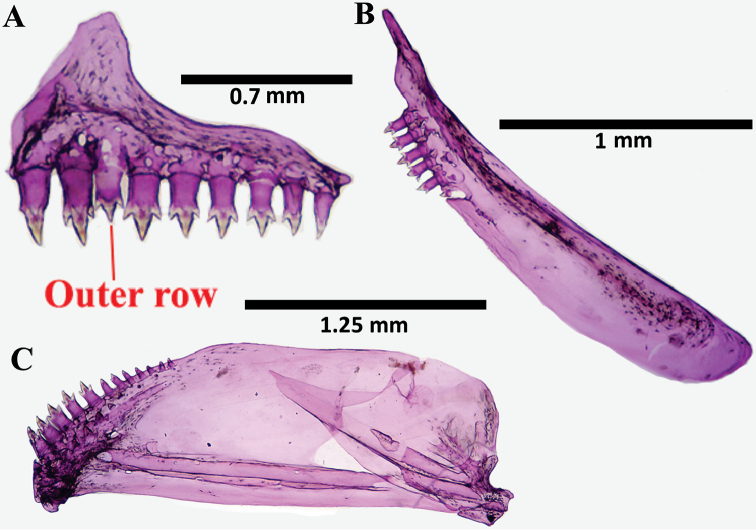
*Hyphessobrycon piorskii* sp. n. CICCAA 00697, 19.3 mm SL; jaw suspensory: **A** Premaxillary. **B** Maxilla **C** Dentary (Photographed by Erick Guimarães).

**Figure 4.**
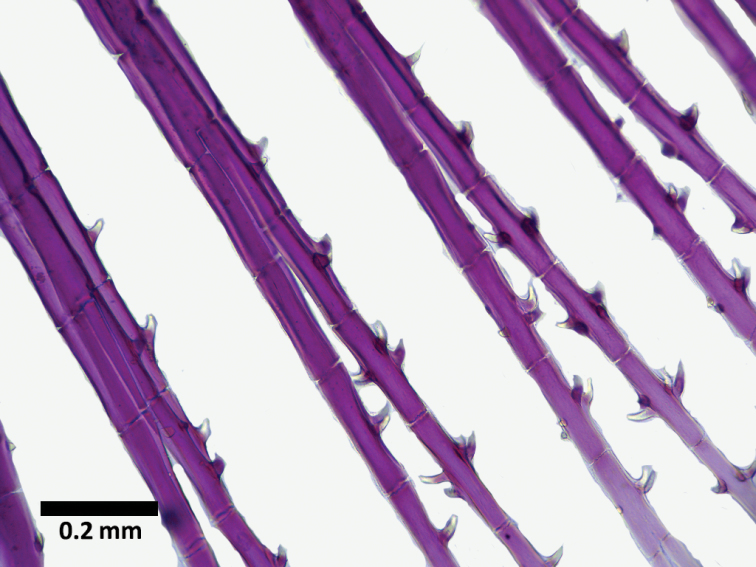
*Hyphessobrycon piorskii* sp. n. CICCAA 00697, male, 19.3 mm SL, bony hooks on anal fin (photographed by Erick Guimarães).

**Figure 5.**
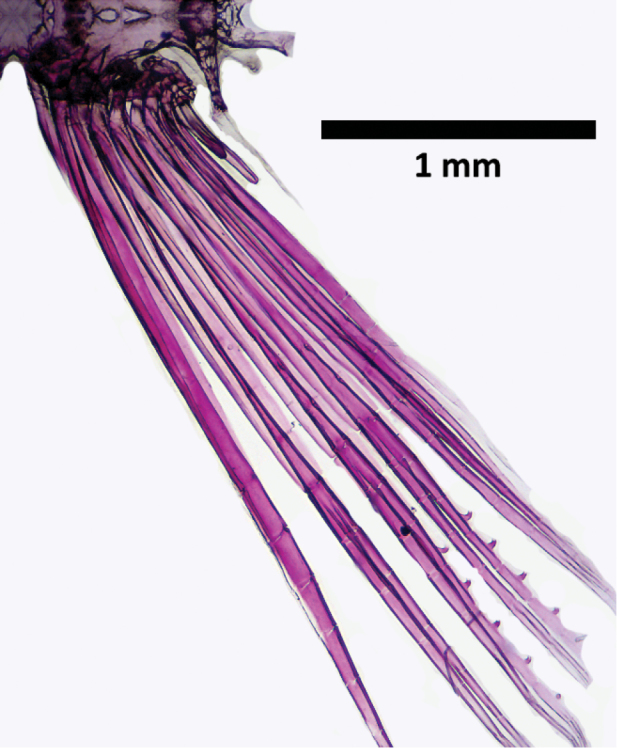
*Hyphessobrycon piorskii* sp. n. CICCAA 00697, male, 19.3 mm SL, bony hooks on pelvic fin (photographed by Erick Guimarães).

Morphometric data of holotype and paratypes are presented in Table 3. Body compressed, moderately deep, greatest body depth slightly anterior to dorsal-fin base. Body profile straight and downward directed from end of dorsal fin to adipose fin, straight or slightly convex between later point and origin of dorsal most procurrent caudal-fin ray. Dorsal profile of head convex from upper lip to vertical through eye; predorsal profile of body roughly straight, dorsal-fin base slightly convex, posteroventrally inclined; ventral profile of head convex from lower jaw to pelvic-fin origin. Ventral profile of body straight or slightly convex from pelvic-fin origin to anal-fin origin; straight and posterodorsally slanted along anal-fin base; and slightly concave on caudal peduncle. Jaws equal, mouth terminal, anteroventral end of dentary protruding. Maxilla reaching vertical to anterior margin of pupil. Premaxillary teeth in two rows. Outer row with one tricuspid tooth; inner row with 6(6), 7(20) or 8(4) tricuspid teeth and one unicuspid tooth. Maxilla with 3(5), 5(24) or 6(1) tricuspid teeth. Dentary with five (21) or six (9) larger tricuspid teeth followed by one smaller tricuspid teeth 5(2), 6(6), 7(13), 8(5), 9(4) smaller unicuspid teeth (Figure 3). Scales cycloid, three to eight radii strongly marked, *circuli* well-marked anteriorly, weakly-marked posteriorly; lateral line incompletely pored, with 6(19), 7(62) or 8(13) perforated scales. Longitudinal scales series including lateral-line scales 31(9), 32(34), 33(26), 34(17) or 35(3). Longitudinal scales rows between dorsal-fin origin and lateral line 6(49) or 7(41). Horizontal scale rows between lateral line and pelvic-fin origin 4(18) or 5(71). Scales in median series between tip of supraoccipital spine and dorsal-fin origin 8(6), 9(14), 10(7) or 11(3). Circumpeduncular scales 11(16), 12(38) or 13(11). Dorsal-fin rays i + 10(105) or ii + 10(18). First dorsal-fin pterygiophore main body located behind neural spine of 4^th^ vertebrae. Adipose fin present. Anteriormost anal-fin pterygiophore inserting posterior to haemal spine of 11^th^ vertebrae. Anal-fin ii+24(3), iii+24(87), ii-25(32) or iii+25(1). Anterior anal-fin margin slightly convex, with anteriormost rays more elongate and slightly more thickened than remaining rays, forming a distinct lobe. Remaining rays smaller with straight distal margin. Anal-fin rays with a sexually dimorphic pattern, which are absent in females (Figure 4). Pectoral fin-rays 12(122) or 13(1) total rays. Tip of pectoral fin usually reaching vertical through pelvic-fin origin. Pelvic-fin rays 8(125) total rays. Pelvic-fin rays with a sexually dimorphic pattern, which are absent in females (Fig. 5). Caudal fin forked, upper and lower lobes similar in size. Principal caudal-fin rays 10+9(121), 10+10(7) or 11+10(17); dorsal procurrent rays 7(1), 9(13), 10(13) or 11(3) and ventral procurrent rays 6(1), 7(8), 8(12) or 9(9). Branchiostegal rays 4(30). First gill arch with 1(1), 2(29) hypobranchial, 11(1), 12(28) or 13(1) ceratobranchial, 1(30) on cartilage between ceratobranchial and epibranchial, and 5(1) or 6(16) epibranchial gill-rakers. Supraneurals 3(2) 4(23) or 5(5). Total vertebrae 29(30).

*Color in alcohol* Figure 1. Ground coloration light yellowish brown. Humeral region with one inconspicuous vertically elongated spot; more intensely pigmented on its central portion. Flank with chromatophores homogeneously scattered, more concentrated on posterior region to humeral spot, posterior region of dorsal-fin base origin and below mid-portion of trunk, between anal-fin origin and caudal peduncle. Ventral region lacking dark brown chromatophores. Dark brown chromatophores present on head and more concentrated on dorsal portion, becoming sparser on cheek and preopercular regions.

Dorsal fin ground coloration hyaline, with a conspicuous black or dark brown spot located on anterior portion of fin, reaching about sixth ray, approximately between half to two thirds of fin depth. Anal and caudal fins hyaline. Caudal fin with a darker, usually dark brown, posterior margin and on its base. Adipose fin hyaline to light brown, with dark brown or black chromatophores more concentrated on its dorsal portion, depending on the state of preservation of the specimen. Pectoral and pelvic fins hyaline; pelvic fin with variable amounts dark brown pigmentation remaining depending on the state of preservation of the specimen.

*Color in life* (Figure 2). Pattern similar to coloration of preserved specimens. Ground coloration light yellowish brown to grey, with a reddish-brown pigmentation on vertebrae region, and usually with red chromatophores. Ventral region anterior to anal-fin origin lighter. Humeral spot inconspicuously dark brown or black. Head with same coloration as body, and ventrally lighter.

Conspicuous black spot on dorsal-fin, with yellow or orange pigmentation on dorsal and ventral margins of spot; yellow or orange pigmentation lighter and less evident on dorsal margin, reaching half to two thirds of the spot length and extending to the tip of fin; yellow or orange pigmentation darker and more developed at ventral margin of the spot, reaching entire spot base length, not extended to dorsal fin-base. Rest of dorsal fin hyaline. Anal-fin base with red pigmentation, with different degrees of intensity, with milk white pigmentation on anterior tip of anal fin, which could be extended through entire anterior margin, reaching between second to fourth rays. Posterior margin of anal fin with an inconspicuous dark brown pigmentation. Adipose fin light brown to hyaline at base, with red to black pigmentation at tip. Pectoral and pelvic fins hyaline, with some sparser dark brown chromatophores, more concentrated at pelvic fin base. First ray of pelvic fin with a white pigmentation. Caudal fin with red pigmentation on almost fin, with an inconspicuous light brown, reddish brown or dark brown margin.

*Sexual dimorphism.* Mature males have hooks on anal-fin and pelvic-fin rays. Hooks absent on females. Anal-fin presenting hooks from 3^rd^, 4^th^ or 5^th^ rays through last ray. Number of hooks variable, increasing from the first ones to the last rays. Pelvic fin presenting 3^rd^ and 4^th^ rays with 5 smaller hooks (Figures 4, 5).

*DNA-based identification.* After trimming sequence ends with poor base call quality, the final alignment yielded 446 base pairs with 154 variable sites, and 22 haplotypes. The magnitude of sequence divergence clearly demonstrates the existence of a new species of *Hyphessobrycon* inhabiting the Munim and Preguiças river basins in Maranhão State. Average genetic distances were 14.2%, with the highest values between *H. pyrrhonotus* and *H. epicharis* (19.2%), while the lowest value (2.7%) was between *H. epicharis* and *H. sweglesi* (Table 1). *Hyphessobrycon piorskii* sp. n. has 17% sequence divergent, on average, from the other taxa, with a minimum distance with *H. eques* (13.9%) and a maximum with *H. rosaceus* (18.4%) (Table 2).

Other evidence for the new species is that *H. piorskii* sp. n. formed a single and exclusive clade with maximum posterior probability support (posterior probability = 1) in the Bayesian phylogenetic tree (Figure 7). Furthermore, *H. piorskii* sp. n. clade is located within the *Hyphessobrycon* *s. str.* group with high support of posterior probability (0.94). *Hyphessobrycon piorskii* sp. n. was recovered as the sister-group of the clade including *H. bentosi*, *H. socolofi*, *H. megalopterus*, *H. erythrostigma* and *H. pyrrhonotus*, with branch support of posterior probability value of 0.55. *Pristella maxillaris* and *Moenkhausia hemigrammoides* formed a clade (posterior probability value = 0.86), and it was recovered as the sister-clade of the *Hyphessobrycon* *s. str.* group (posterior probability value = 0.6).

**Table 2.** Kimura-2 parameters pairwise genetic distances among species. Species names in the upper columns are abbreviated as follows: *H. piorskii* (Hpio), *H. flammeus* (Hfla), *H. anisitsi* (Han), *H. socolofi* (Hsoc), *H. copelandi* (Hcop), *H. bentosi* (Hben), *H. megalopterus* (Hmeg), *H. eques* (Hequ), *H. erythrostigma* (Hery), *H. pyrrhonotus* (Hpyr), *H. rosaceus* (Hros), *H. sweglesi* (Hswe), *H. epicharis* (Hepi), *M. hemigrammoides* (Mhem), and *P. maxillaris* (Pmax).

Species	Hpio	Hfla	Hani	Hsoc	Hcop	Hben	Hmeg	Hequ	Hery	Hpyr	Hros	Hswe	Hepi	Mhem
Hfla	0.190	-												
Hani	0.194	0.165	-											
Hsoc	0.199	0.193	0.168	-										
Hcop	0.158	0.206	0.162	0.216	-									
Hben	0.207	0.204	0.173	0.008	0.221	-								
Hmeg	0.195	0.209	0.180	0.087	0.213	0.086	-							
Hequ	0.161	0.205	0.186	0.190	0.102	0.194	0.199	-						
Hery	0.196	0.213	0.182	0.114	0.191	0.117	0.131	0.174	-					
Hpyr	0.183	0.206	0.166	0.101	0.191	0.103	0.117	0.181	0.032	-				
Hros	0.218	0.212	0.205	0.224	0.202	0.227	0.204	0.195	0.219	0.221	-			
Hswe	0.205	0.187	0.195	0.224	0.198	0.229	0.192	0.183	0.216	0.211	0.073	-		
Hepi	0.199	0.190	0.198	0.221	0.198	0.226	0.199	0.183	0.233	0.231	0.089	0.028	-	
Mhem	0.185	0.204	0.178	0.214	0.216	0.219	0.234	0.205	0.233	0.222	0.200	0.204	0.208	-
Pmax	0.223	0.212	0.197	0.203	0.219	0.207	0.218	0.200	0.230	0.239	0.202	0.187	0.181	0.169

**Table 3.** Morphometric data (N = 95) for the holotype and paratypes of *Hyphessobrycon piorskii* sp. n. from the Munim River basin and Preguiças River basin. Abbreviations: SD: Standard deviation.

	Holotype	Paratypes	Mean	SD
Standard length	25.9	18–29.4	20.8	–
**Percentages of standard length**				
Depth at dorsal-fin origin (body depth)	35.9	28.9–39.4	33.4	1.9
Snout to dorsal-fin origin	49.8	44.2–56.5	52.5	2.1
Snout to pectoral-fin origin	29.1	26.0–35.0	30.8	2.3
Snout to pelvic-fin origin	47.5	39.1–52.2	47.5	1.9
Snout to anal-fin origin	61.9	57.4–66.6	61.7	1.8
Caudal peduncle depth	11.8	9.1–14.1	11.4	0.9
Caudal peduncle length	12.2	8.1–13.6	10.2	1.1
Pectoral-fin length	20.4	16.8–23.7	20.8	1.6
Pelvic-fin length	18.1	13.3–20.4	17.1	1.5
Dorsal-fin base length	15.5	12.9–18.3	15.7	1.2
Dorsal-fin height	28.5	22.1–34.3	29.9	2.4
Anal-fin base length	29.4	26.3–33.9	30.3	1.4
Eye to dorsal-fin origin	35.2	33.6–39.4	36.4	1.4
Dorsal-fin origin to caudal-fin base	50.7	44.9–57.3	51.4	2.3
Head length	26.3	24.2–33.4	29.3	2.1
**Percentages of head length**				
Horizontal eye diameter	42.8	33.4–43.8	38.2	2.3
Snout length	22.0	16.9–24.4	20.2	1.7
Least interorbital width	25.7	16,4–27.0	20.4	2.2
Upper jaw length	39.6	32.8–41.7	38.1	2.2

#### Geographical distribution.

*Hyphessobrycon piorskii* sp. n. is presently known only from the upper Munim and Preguiças river basins, Maranhão State, northeastern Brazil (Figure 7).

#### Ecological notes.

*Hyphessobrycon piorskii* sp.n. lives in shallow well-oxygenated streams with transparent waters flowing over different types of substrates (Figure 8). The streams where *H. piorskii* sp. n. specimens were collected varied from 0.90 to 10 meters wide, with a maximum depth of 1.60 meters. They possessed moderate water currents (0.1–0.7 m/s), with clear, sandy substrates with pebbles, mud, leaf litter, and submerged logs, often also presenting aquatic macrophytes. *Hyphessobrycon piorskii* sp. n. was found near shore among aquatic vegetation, tree roots and fallen logs. Other species found at both sites were *Anablepsoides vieirai* Nielsen, 2016, *Apistogramma piauiensis* Kullander, 1980, *Astyanax* sp., Cichlasoma cf. zarskei, *Copella arnoldi* (Regan, 1912), *Crenicichla brasiliensis* (Bloch, 1792), *Hoplias malabaricus* (Bloch, 1794), *Megalechis thoracata* (Valenciennes, 1840), *Nannostomus beckfordi* Günther, 1872, and *Synbranchus marmoratus* Bloch, 1795. Gut contents of C&S specimens contained algae and disarticulated arthropod remains.

#### Etymology.

The name *piorskii* honors the ichthyologist Nivaldo Magalhães Piorski for his contributions to the ichthyologic knowledge of the Maranhão State.

## Discussion

Despite *Hyphessobrycon*, as defined today, being a non-monophyletic group (Mirande 2010, Oliveira et al. 2011, Carvalho et al. 2017, Ohara et al. 2017, Moreira and Lima 2017), a few putative groups within the genus were proposed in the literature. One such case is the *Hyphessobrycon* *sensu stricto* as defined by Carvalho (2011) and Cavalho and Malabarba (2015). According to those authors, this group is composed by approximately 25 species.

**Figure 6.**
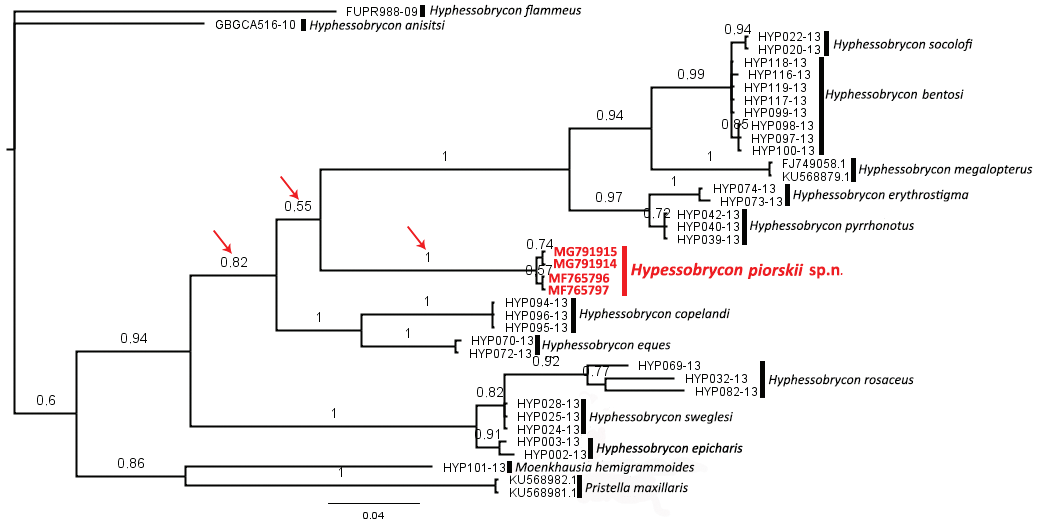
Bayesian phylogenetic tree including *Hyphessobrycon piorskii* sp. n (in red) and other congeners. Number above branches are posterior probability values.

Among the species considered by Weitzman and Palmer (1997) as possibly related to the “rosy tetra clade”, only *H. hasemani* and *H. pulchripinnis* were considered to belong to *Hyphessobrycon* *sensu stricto* (Carvalho 2011, Carvalho and Malabarba 2015). *Pristella maxillaris* (Ulrey, 1894) is the sister-group of the *Hyphessobrycon* *sensu stricto* (Carvalho 2011), and corroborated in our analysis (Figure 6).

*Hyphessobrycon piorskii* sp. n. exhibits all the diagnostic features that define *Hyphessobrycon* *sensu stricto* (see introduction and diagnosis section). The new species differs from the other possible species of this assemblage, which also occur near Maranhão (e.g., lower Amazon River basin, Guamá River basin, and São Francisco River basin), such as *H. bentosi, H. copelandi, H. eques, H. dorsalis, H. hasemani, H. haraldschultzi, H. micropterus*, and *H. werneri*, by a set of features listed below.

*Hyphessobrycon piorskii* sp. n. possesses an inconspicuous vertically elongated humeral spot, distinguishing it from all the species cited above, except for *H. bentosi* and *H. hasemani* (see morphological diagnosis section). The shape of the dorsal-fin spot is also useful to distinguish *H. piorskii* sp. n. from *H. eques, H. hasemani* and *H. micropterus*, which possess dorsal fin spot vertically extended, reaching the tip of the fin, while in *H. piorskii* sp. n. the black spot of dorsal fin never reaches the tip of the fin. The new species also differs from *H. eques* by the color pattern of the anal fin: *H. eques* possess a conspicuous black anal-fin margin on preserved species, while *H. piorskii* sp. n. does not exhibit this feature at the anal fin.

The number of teeth cusps was also revealed to be a useful feature for species discrimination. *Hyphessobrycon piorskii* sp. n. possess all of its teeth with one to three cusps (never pentacuspid), while *H. eques* possess pentacuspid teeth on the maxillary and inner row of premaxillary, and *H. copelandi* and *H. hasemani* on the dentary and inner row of the premaxilla (see Lima et al. 2013). The new species differs from *H. bentosi* by not having extended and pointed dorsal and anal-fin tips and by having bone hooks on anal-fin rays of mature males (Figure 3). The dorsal and anal fins of *H. bentosi* have pointed and extended tips, and it has not bony hooks on anal-fin rays (see Carvalho 2011, Zarske 2014). *Hyphessobrycon copelandi* possesses only ten teeth on the dentary, and dorsal-fin black spot reaching to the posterior margin of the fin (see Lima et al. 2013), while *H. piorskii* sp. n. possesses 11–15 teeth on dentary, and dorsal-fin black spot restricted to the anterior half of the fin’s length. In addition, *Hyphessobrycon piorskii* sp. n. is easily distinguished from the sister-species of the clade *Hyphessobrycon* *sensu stricto*, *P. maxillaris* and *M. hemigrammoides*, by the absence of a black oblique stripe or band on the anterior portion of the anal fin (Figures 1, 2) [vs. presence (Carvalho et al. 2017, figure 7; pers. obs.)].

The description of *H. piorskii* sp. n. was based on morphological and molecular species delimitation methods, using the congruence of multiple operational criteria for determining species boundaries. As mentioned earlier, evidence corroborated from multiple operational criteria is considered to produce stronger hypotheses of lineage divergence (de Queiroz 2007, Goldstein and Desalle 2010), thus congruent to the proposal for an integrative taxonomy (Goldstein and Desalle 2010, Padial et al. 2010). The morphological criteria (PAA) distinguished the new species from all of the other congeners by unambiguous character states (see diagnosis). The DNA barcoding (DBC) criteria also revealed that *H. piorskii* sp. n. is a new species with an average sequence divergence of 17% from the other taxa (Table 2). In addition, *H. piorskii* sp. n. is placed in an exclusive and highly supported clade in the Bayesian tree (Figure 6). Haplotypes clustered as an exclusive and high supported group, with geographical concordance area is evidence of lineage divergence, therefore a good and strong evidence for delimit species, and consequently describe them (Wiens and Penkrot 2002, Costa et al. 2014).

**Figure 7.**
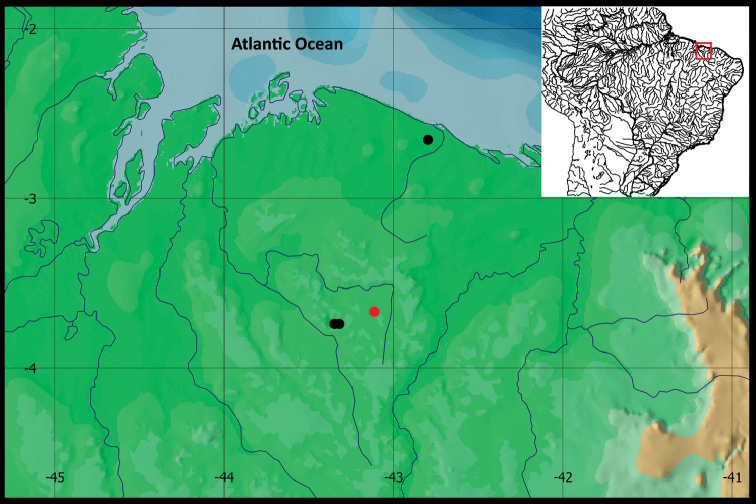
Geographical distribution of *Hyphessobrycon piorskii* sp. n. Red circle denote Holotype and black circle denote paratypes.

Our Bayesian tree also recovered *H. piorskii* sp. n. within the *Hyphessobrycon* *sensu stricto* group with high support (posterior probability = 0.94), which fits the morphological evidence, since *H. piorskii* sp. n. exhibits the three main diagnostic character states of the group (see introduction and diagnosis section). *Hyphessobrycon piorskii* sp. n. was recovered as the sister-group of the clade including *H. bentosi*, *H. socolofi*, *H. megalopterus*, *H. erythrostigma*, and *H. pyrrhonotus*, however this relationship was supported by a lower support value (posterior probability value = 0.55). Only posterior probability values about or higher than 0.95 are considered as statistically significant (Alfaro and Holder 2006). Therefore, any discussion about the relationship and supposed shared morphological features between *H. piorskii* sp. n. and this clade is speculative (Figure 7). To a better understanding of the internal relationships of the group, an analysis including more genes, especially from nuclear genome, is highly recommended. However, this was not the scope of the present paper. *Pristella maxillaris* and *Moenkhausia hemigrammoides* were recovered as the sister-clade of the *Hyphessobrycon* *sensu stricto* group, corroborating partially the results of Carvalho et al. (2011) and Carvalho and Malabarba (2015), who argue that *P. maxillaris* is the sister-clade of the *Hyphessobrycon* *sensu stricto* group.

**Figure 8.**
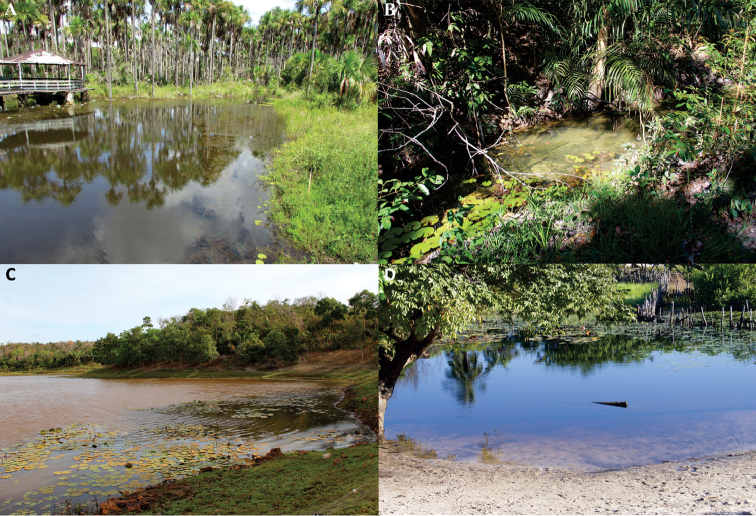
Collecting sites of *Hyphessobrycon piorskii* sp. n. **A** stream at the Anapurus municipality **B** stream at Mata de Itamacaoca **C** stream at Mata de Itamacaoca **D** stream at Mata Fome, Barreirinhas municipality (photographed by Felipe Ottoni).

## Supplementary Material

XML Treatment for Hyphessobrycon piorskii
